# Normal Variants and Pitfalls of ^18^F-FDG PET/CT Imaging in Pediatric Oncology

**DOI:** 10.3389/fnume.2022.825891

**Published:** 2022-03-03

**Authors:** Khushica Purbhoo, Mboyo Di-Tamba Vangu

**Affiliations:** Department of Nuclear Medicine and Molecular Imaging, Chris Hani Baragwanath Academic Hospital, University of the Witwatersrand, Johannesburg, South Africa

**Keywords:** ^18^F-FDG PET/CT, pediatric nuclear medicine, oncology, physiological, normal variant

## Abstract

Positron emission tomography (PET) with 2-[fluorine-18] fluoro-2- deoxy-D-glucose (FDG) is a well-established modality that is used in adult oncologic imaging. Its use in pediatric oncology has increased over time. It enables increased diagnostic accuracy due to the combination of functional and morphologic imaging, resulting in optimal patient management. However, the clinician should be aware that the normal distribution of FDG uptake in children differs from adults. Also, even though FDG is used widely in oncology, it is not tumor specific. Uptake of FDG may be seen in numerous benign conditions, including inflammation, infection, and trauma. Proper interpretation of pediatric FDG PET/CT studies requires knowledge of the normal distribution of FDG uptake in children, and an insight into the physiologic variants, benign lesions, and PET/CT related artifacts. Understanding the potential causes of misinterpretation increases the confidence of image interpretation, reduce the number of unnecessary follow-up studies, optimize treatment and more importantly, reduce the radiation exposure to the patient. We review and discuss the physiological distribution of FDG uptake in children, the variation in distribution, lesions that are benign that could be misinterpreted as malignancy, and the various artifacts associated with PET/CT performed in pediatric oncology patients. We add a pictorial illustration to prompt understanding and familiarity of the above-mentioned patterns. Therefore, we believe that this review will assist in reducing possible mistakes by reading physicians and prevent incorrect interpretation.

## Introduction

Positron emission tomography (PET) with 2-[fluorine-18] fluoro-2- deoxy-D-glucose (FDG) is a well-established modality that is used in adult oncologic imaging. Its use in pediatric oncology has increased over time. It enables increased diagnostic accuracy due to the combination of functional and morphologic imaging, resulting in optimal patient management. However, the clinician should be aware that the normal distribution of FDG uptake in children differs from adults (1). Also, even though FDG is used widely in oncology, it is not tumor specific. Uptake of FDG may be seen in numerous benign conditions, including inflammation, infection and trauma. Proper interpretation of pediatric FDG PET/CT studies requires knowledge of the normal distribution of FDG uptake in children, and an insight into the physiologic variants, benign lesions, and PET/CT related artifacts. Understanding the potential causes of misinterpretation increases the confidence of image interpretation, reduce the number of unnecessary follow-up studies, optimize treatment and more importantly, reduce the radiation exposure to the patient.

We review and discuss the physiological distribution of FDG uptake in children, the variation in distribution, lesions that are benign that could be misinterpreted as malignancy, and the various artifacts associated with PET/CT performed in pediatric oncology patients. We add a pictorial illustration to prompt understanding and familiarity of the above-mentioned patterns. Therefore, we believe that this review will assist in reducing possible mistakes by reading physicians and prevent incorrect interpretation.

## ^18^F-FDG Molecule

FDG is an analog of glucose and is labeled with a positron-emitting isotope, F (fluorine)−18. It is transported into the cells by glucose transporters (GLUT), is phosphorylated by hexokinase and remains trapped within the cell (1, 2). Cancer cells preferentially use non-oxidative glucose metabolism with up-regulation of glucose transporter receptors and hexokinase and a reduced intracellular glucose-6-phosphatase expression. This results in accumulation of FDG within the tumor cells at a greater rate than in normal tissue. Active inflammatory processes also cause an increase in FDG uptake. This is due to increased glucose use by activated granulocytes and mononuclear cells (3, 4). Thus, FDG is not tumor specific and accumulates in non-malignant pathology, such as infection and inflammation (5–7).

## Oncologic Indications For Imaging With ^18^F- FDG

The most common indications for FDG PET imaging in pediatric oncology includes lymphoma (Hodgkin's disease and non- Hodgkin's lymphoma), bone and soft tissue sarcoma, neuroblastoma, and central nervous system tumors (1). The most widely researched indications for FDG PET/CT in lymphoma are for staging, restaging and evaluation of the response to therapy, assessment of residual mass after therapy, biopsy, and radiation therapy planning (2).

## Contraindications

Although uncommon in the pediatric age group, the possibility of pregnancy in female patients of child-bearing age should always be considered. Last menstruation period screening of female patients of child-bearing age is essential before scheduling the study. Screening typically begins at 10 years and older (8, 9). The decision to carry out the study is balanced against the risk of carrying out the scan.

## Patient Preparation

Crucial information is required for optimal interpretation of FDG PET/CT images, such as clinical history, including the type of suspected or known tumor; current symptoms; the results of previous imaging; timeline in terms of surgery, chemotherapy, or radiation therapy and a history of recent infection/inflammation (1, 2). A thorough explanation and written instruction of the procedure should be shared with the patient and his/her parents/guardian (1, 2). At our institution the patient/parent/guardian is called 24–48 h prior to the scan to discuss the preparation for the study. Written information is also given at the time of booking the study. The patient should fast for at least 4–6 h prior the study, but can drink water to maintain good hydration, should sedation not be considered (1, 2). Intravenous hydration post FDG injection, during the uptake period can be achieved with 0.9% saline solution. Soft drinks and sweets during the preparation phase are strongly discouraged (1).

The radiotracer injection should be timed as close as possible to breast/bottle feed in infants and a feed may be given from 30 min after the injection (1, 2). Prior to the injection, the fasting blood glucose level should be determined, with the preferred level being lower than 11.1 mmol/L (200mg/dL) (1). High blood glucose level result in circulating glucose that competitively inhibits the cellular uptake of FDG and decreases FDG uptake at the sites of pathology. Patients without diabetes mellitus may develop hyperglycemia when stressed or after starting glucocorticoid therapy. The PET study should be delayed until these patients have better control of their glucose. Metformin has been shown to cause intense FDG uptake in the colon and in the small intestine (4). It also increases the skeletal muscle and liver uptake of FDG. Therefore, if not contraindicated, metformin should be discontinued 24–48 h before the study (4). The supervising physician should be notified if the blood glucose level is >11.1 mmol/L, and a decision should be made whether to proceed with the FDG injection (1).

To reduce the discomfort caused by the intravenous cannula, local anesthetic creams can be used. The duration of the uptake period should be kept constant with a <10% variation if studies are being compared to allow for comparison of standard uptake values (4). Ideally, the uptake time should be between 60 and 90 min (1). The patient should remain in a quiet and warm environment during the uptake period. With the advent of electronic devices, patients may want to utilize them, however, the repetitive hand motion required to operate these devices increases the FDG uptake in the involved muscles (4).

**Figure 1:**
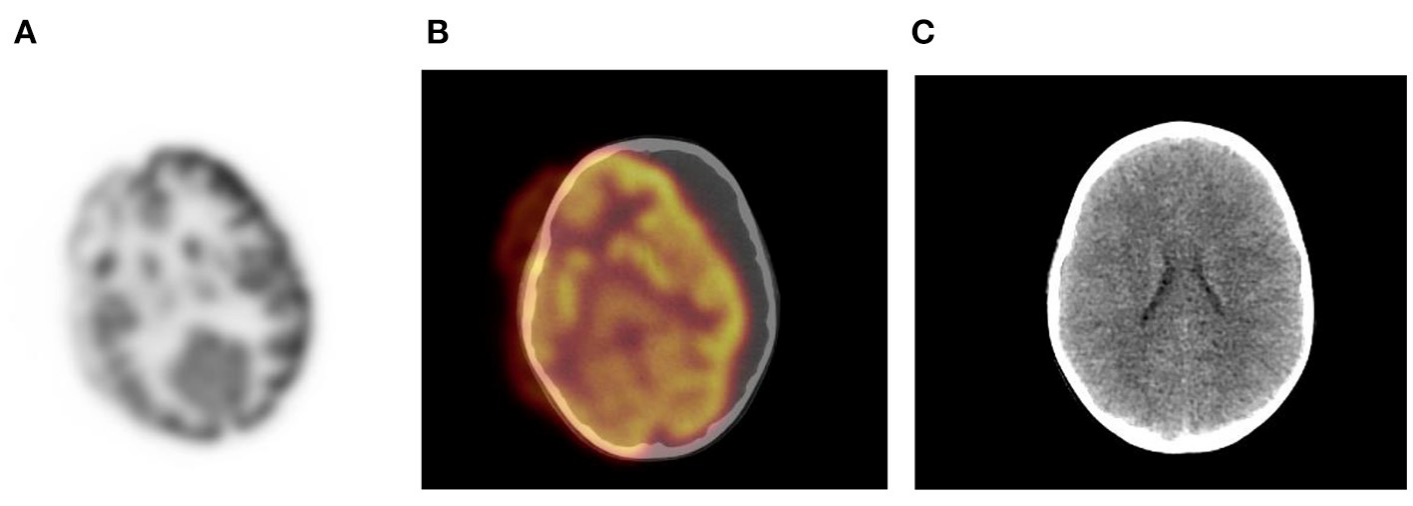
Motion artifact of the brain on axial PET and PET/CT images **(A,B)**. No obvious pathology is seen on the non- contrast axial CT component **(C)** of the PET study.

CT protocols and intravenous (IV) contrast should be adjusted to the body weight to decrease the radiation dose (10). The principle of ALARA (As Low as Reasonably Achievable) should always be followed at all times. Optimal tube voltage and mAs should be used to reduce the radiation dose (10). A dose of 6 MBq/kg body weight FDG in 2D mode scanning acquisition and 3 MBq/kg body weight in 3D mode scanning acquisition is recommended for pediatric patients (1, 2). After injection, the child should avoid talking, chewing or excessive motion. During the uptake phase children should be kept warm, preferably in a warm environment with the use of blankets. This reduces radiotracer uptake in thermogenic brown fat. The need for a sedative must be individualized per patient and considered in children who cannot remain motionless in the scanner for at least 15–20 min. Positioning with immobilizing devices will help to reduce mis-registration artifacts and mitigate repeating imaging acquisition (Figure 1). Generally, sedation protocols are performed in accordance with departmental guidelines. Irritable, uncooperative or claustrophic children will most likely need sedation (1).

Bladder catheterization can reduce the accumulation of tracer activity in the bladder. Insertion of urinary catheter is not routine practice in pediatric patients as it may increase the risk of infection and cause added stress for the child (2). Children who are investigated for tumors in the pelvis may be considered for catheterization to avoid lesions being obscured by tracer activity in the urinary bladder. This will also reduce the radiation dose to the child (2). Furosemide (0.5–1 mg/kg; maximum dose, 20 mg) can be given at the time of injection or 15 min post injection to enhance diuresis and minimize activity in the urinary tract (2). The child should be encouraged to void prior to the start of acquisition. In infants who are not toilet trained, the nappy should be changed before acquisition (1).

## Acquisition

The patient should be comfortably immobilized during the study acquisition with Velcro straps, tapes, or cushions to avoid movement artifacts (1). A field of view extending from the skull base to the mid-thigh is routinely used. The added radiation exposure to the pediatric patient from the CT component of the study has been of concern (1, 2). In neuroblastoma and sarcoma patients and in patients with lymphoma who have suspected bone or bone marrow disease, the entire upper limb and lower limbs should be included in the field of view (1).

Premedication to minimize brown adipose tissue or muscle uptake is used. Oral contrast increases the attenuation artifact that may result from mis-registration between the CT and PET acquisition. This risk is greatest with barium containing oral contrast agents and is lower with negative contrast agents, such as water (4).

## Normal Bio-Distribution of FDG

**Figure 2:**
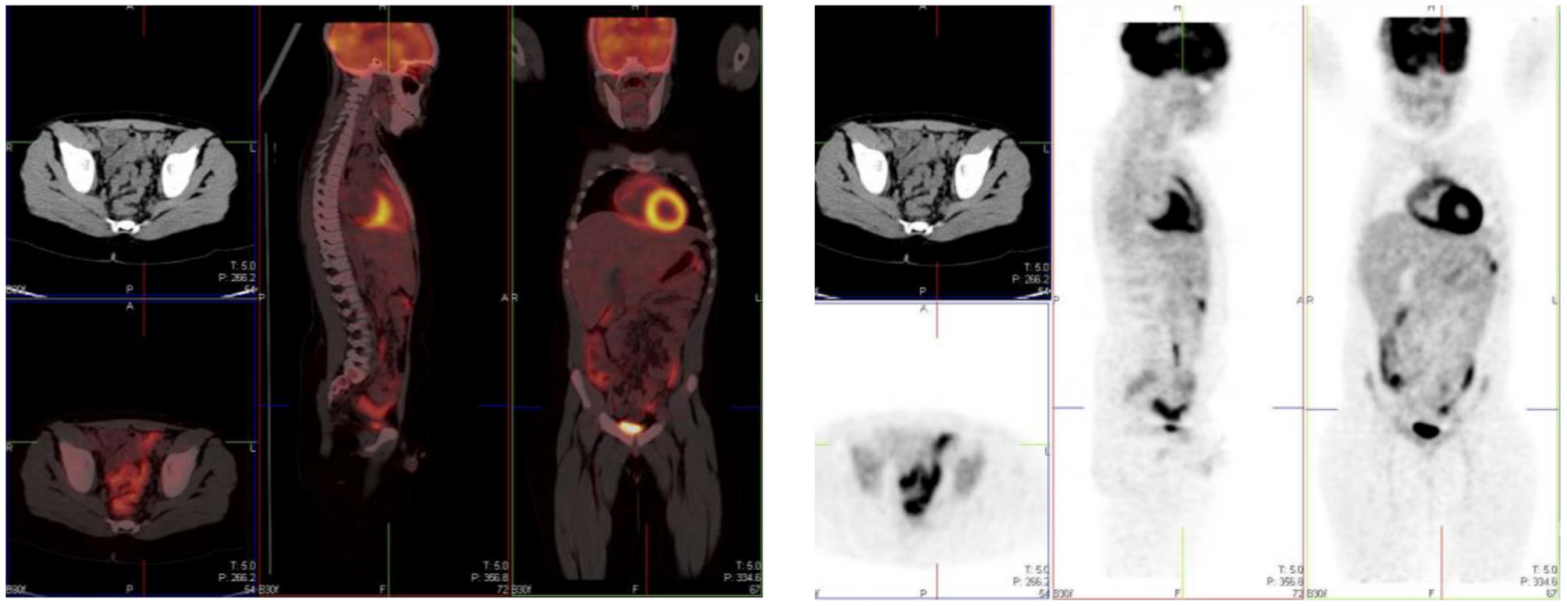
Physiologic uptake of ^18^F-FDG. Axial, sagittal and coronal FDG PET/CT **(left)** and PET images **(right)** show physiologic uptake in a 13 year old boy. Normal FDG uptake is seen in the brain, heart, liver, spleen, colon, urinary bladder and the bone marrow. Note the marked intense activity in the brain compared with the activity in the rest of the body.

The normal bio-distribution and physiologic variants of FDG uptake in children differ from adults, and it is important to recognize these to avoid misinterpretation. The physiologic accumulation of FDG mimics glucose metabolism, which is variable between patients (Figure 2). Physiologic uptake is normally seen with intense uptake in the brain. Uptake is also seen in the heart, liver, spleen, gastrointestinal tract, bone marrow, and there is excretion from the renal collecting system into the ureters and urinary bladder.

## Head And Neck

The brain is dependent on glycolytic metabolism as a source of energy and physiological uptake is seen in the normal cerebral cortex and basal ganglia (Figure 2). During the fasted state, the brain metabolism accounts for 20% of whole-body metabolism (1). The injected dose of FDG represents 6% of the total brain uptake (2). In infants, the glucose uptake differs from older children (4). FDG uptake is most intense in the gray matter and basal ganglia in older children and neuronal activation increases FDG uptake (4). Uptake in malignant primary tumors may be obscured by the intense FDG uptake in brain and metastases in the brain parenchyma adjoining scalp and skull bones may be missed. It is essential to window the gray scale appropriately in order to not miss a lesion in the skull and the brain.

**Figure 3:**
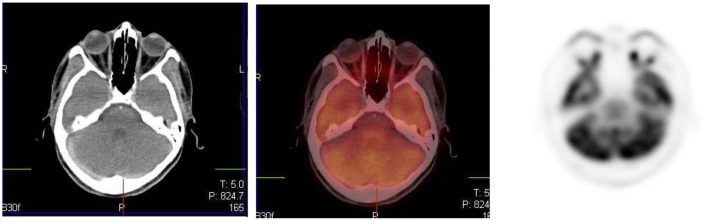
Axial CT, fused PET/CT and PET only images showing physiologic uptake in the medial and lateral ocular muscles.

Uptake in the visual cortex in the occipital lobe due to visual activation will occur if a child is kept in a room with bright lights during the uptake period. Patients should be kept in a dimly lit room with minimal sensory stimulation during the uptake phase to minimize this effect. Muscle activity can be seen at the convergence of the extraocular muscles and along the length of these muscles (Figure 3).

**Figure 4:**
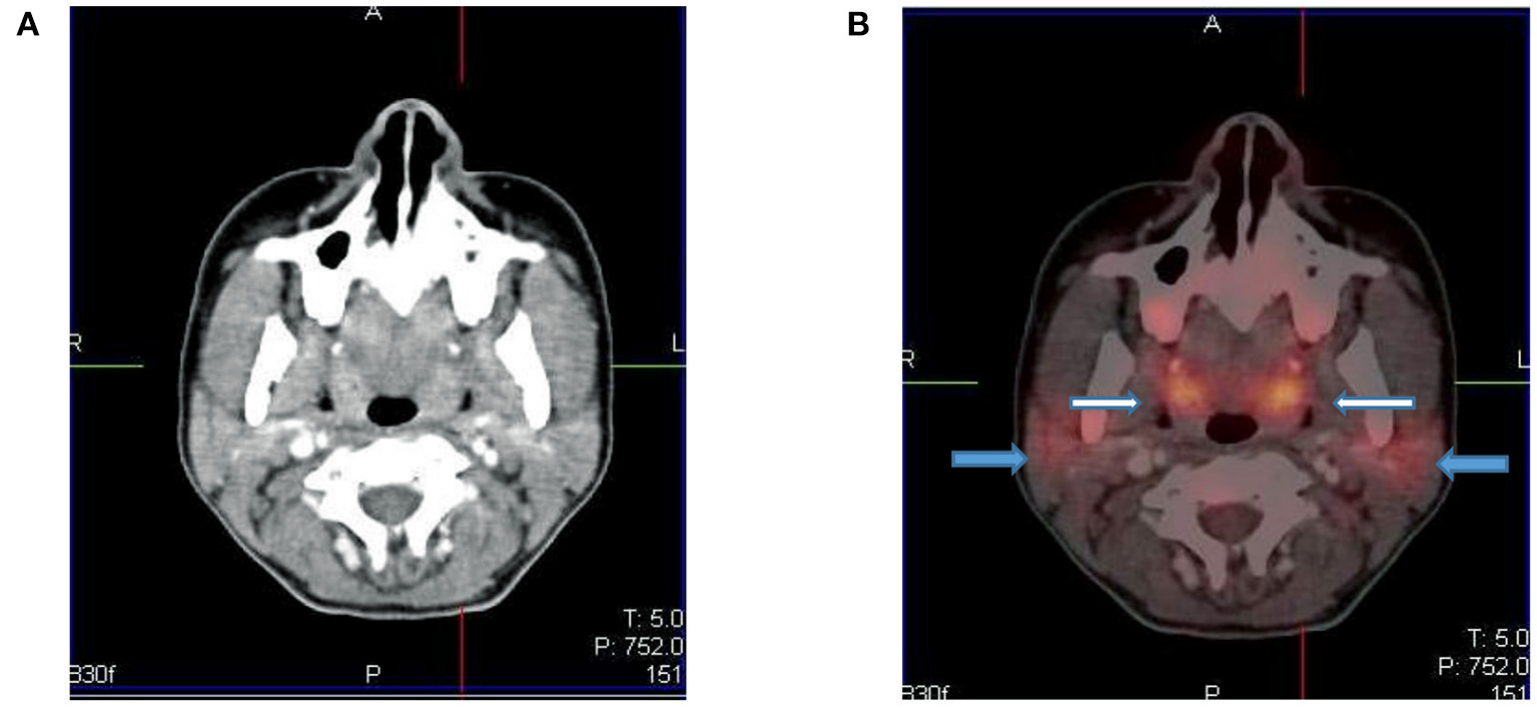
Axial CT **(A)** and fused PET/CT **(B)** images showing symmetric increased uptake in the tonsils (white arrows) and bilateral mild uptake in the parotid glands (blue arrows).

The Waldeyer's ring is consists of lymphoid tissue in the nasopharynx and oropharynx. It consists of adenoids (nasopharyngeal tonsils), palatine tonsils faucial tonsils and lingual tonsils at the base of the tongue and the lateral aspect of the oropharynx. It shows intense FDG uptake in 6–8 years of age and diminishes with age (10). Underlying pathology may be masked by the intense FDG uptake in the Waldeyer's ring. Symmetrical uptake is likely physiologic and asymmetrical uptake may be pathological (1, 4). Mild to moderate uptake can be seen in the adenoids, tonsils, and at the base of the tongue due to the physiologic activity of lymphatic tissue (Figure 4). Infants may show increased uptake in the oral muscles due to sucking of a pacifier during the uptake phase (2, 3).

The larynx and vocal cords show either no uptake or mild symmetrical uptake, which may have an inverted U shape. Prominent laryngeal uptake that is symmetrical is often observed in smaller children if they cry after tracer injection (1). It is important to remind children not to talk during the uptake phase, as excessive talking may cause prominence of activity in the laryngeal structures. Asymmetric uptake in the vocal cord is suggestive of pathology such as malignancy, post-therapy change, or unilateral vocal cord paralysis. Diffusely increased uptake in the salivary gland can be seen after chemotherapy or radiation therapy (1).

FDG uptake in the thyroid gland is rare in children. Diffuse thyroid uptake may represent Graves' disease or thyroiditis. Focal uptake in the thyroid gland is seen in benign thyroid nodules or thyroid malignancy, and further work-up is suggested in such scenario.

**Figure 5:**
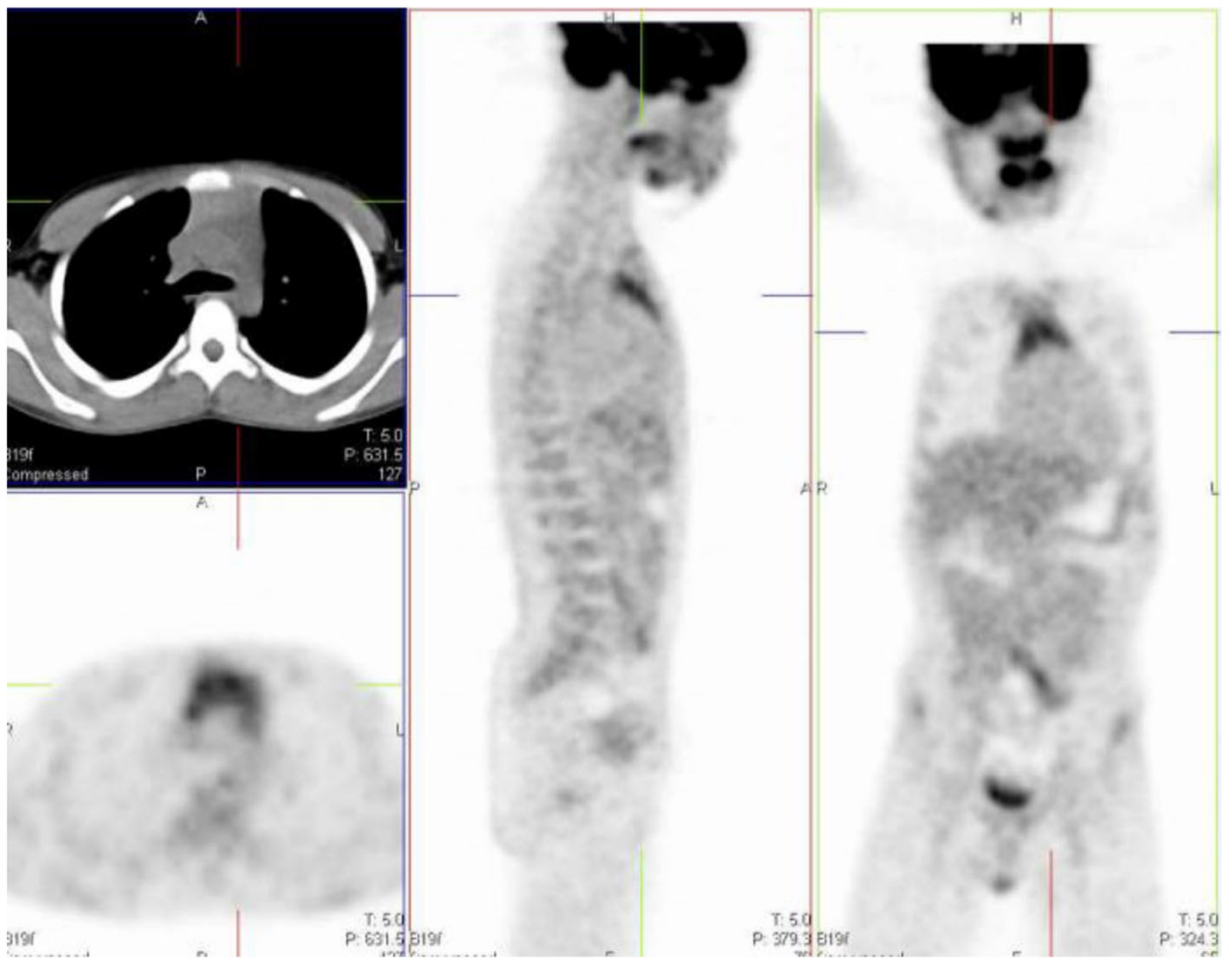
12 year old male referred for the evaluation of active vasculitis. FDG PET study showing diffuse symmetrical increased uptake in the thymus seen on the axial, sagittal and coronal views (inverted ‘'V”).

Homogeneous uptake is seen in the thymus in healthy children (1, 2). The thymus is a lymphoid organ, is bilobed lymphoid organ and is located in the anterior and superior mediastinum and has an inverted ‘V' shape on the coronal plane (Figure 5). The shape and size of thymus is age-related. Neonates have a large thymus, and can increase in size up to the age of two (11). During adolescence there is involution of the thymus and it is replaced with fat, resulting in a decrease of the physiologic uptake. It can cover the left and right aspects of the mediastinum. Prominence of thymic uptake is seen following chemotherapy (2, 12). Homogeneous thymus uptake at post-therapy FDG PET imaging and in the absence of uptake at pre-therapy FDG PET likely indicates post-therapy thymic hyperplasia. Thymic hyperplasia occurs in children during severe stress or chronic disease named “thymic rebound,” when seen after chemotherapy (11). Intense or heterogeneous uptake may raise suspicion for thymus or other anterior mediastinal pathology. If the activity in the thymus equals the uptake in the bladder or cerebellum, it is suggestive of malignancy (12). In a study by Brink et al., increased FDG accumulation in the thymus was seen in 73% of children with a malignancy prior to chemotherapy and in 75% of children post chemotherapy (12). Thus, in children, when evaluating the thymus awareness of the patterns of uptake must be noted. It is helpful to use the CT component of the study as a guide to assist interpretation (13).

## Chest

### Breast

**Figure 6:**
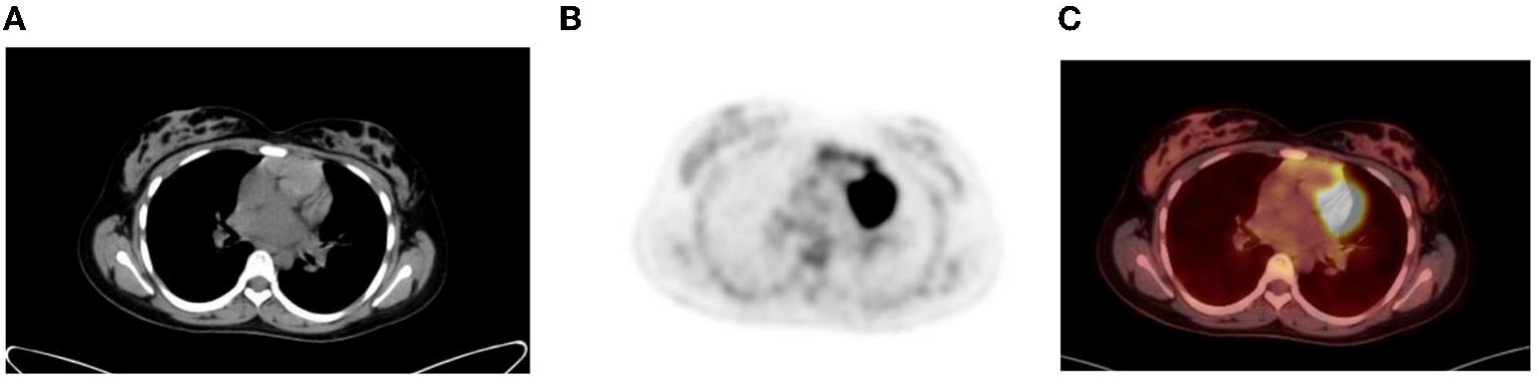
Diffuse uptake in the breast tissue in a 17-year-old female with embryonal rhabdomyosarcoma referred for interim PET study. CT **(A)** PET **(B)** and fused PET/CT **(C)** showing mild low grade uptake in bilateral breast tissue.

The proliferative glandular tissue of the breast results in diffuse physiological FDG uptake (1). Higher uptake may be seen in adolescence due to dense breast tissue. The nipples normally have uptake, and this is identified on non-attenuation-corrected images (Figure 6).

### Myocardium

**Figure 7:**
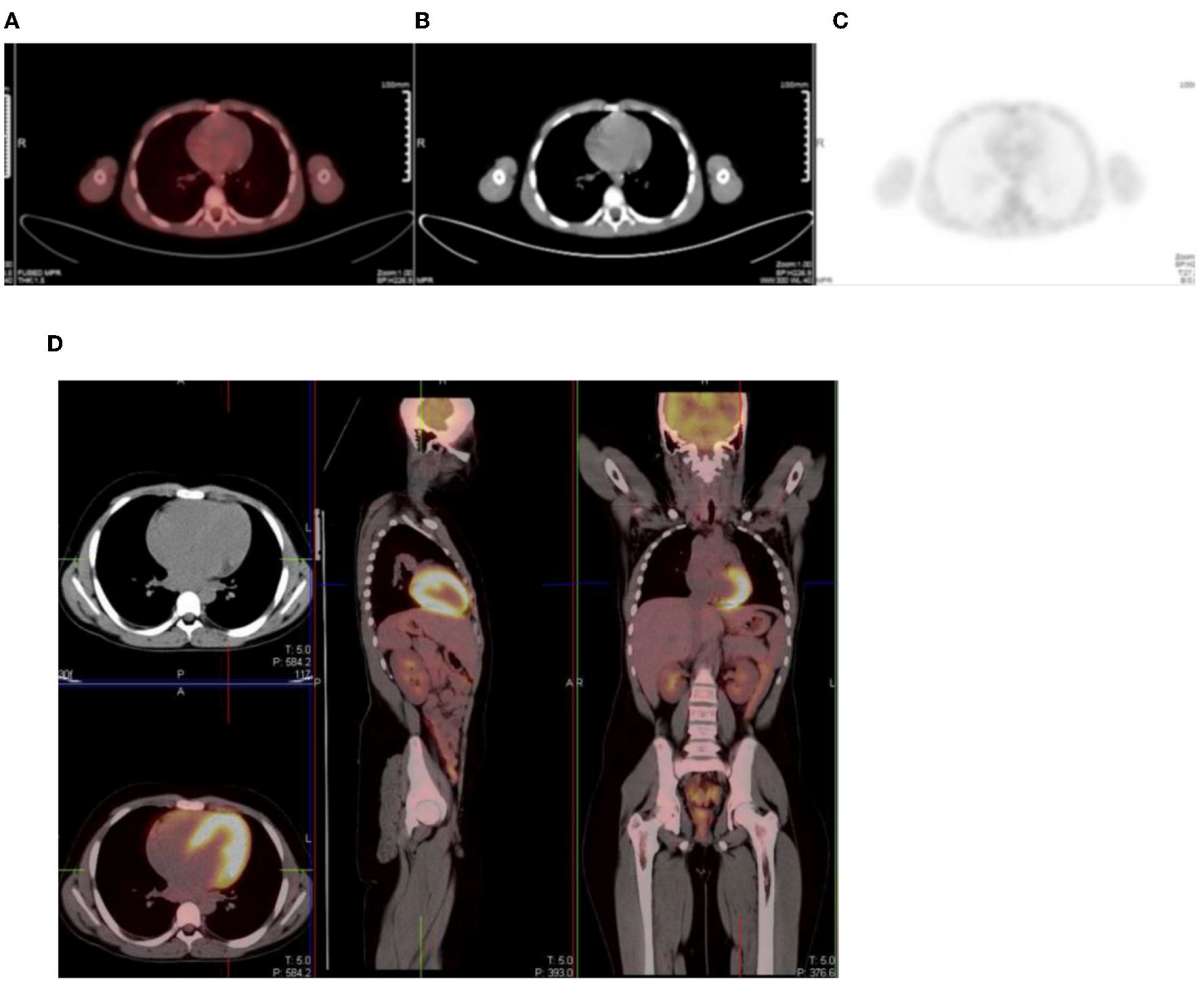
**(A–C)** Axial fused FDG PET/CT **(A)**, CT **(B)** and PET **(C)** images showing a 16 year old male who was well fasted and shows no/minimal cardiac uptake. **(D)** Transverse, sagittal and coronal PET/CT in a 13 year old boy with intense physiological FDG uptake in the left ventricle of the myocardium.

Myocardial uptake is variable and can range from no uptake to intense uptake in the left ventricular myocardium (1). Cardiac uptake depends on substrate availability (2, 14). FDG uptake is low during fasting, as the predominant source of substrate is fatty acids, and the serum insulin level is low (Figures 7A–C). The uptake can be variable or inhomogenous even in the fasting state, and findings may be misinterpreted as a mediastinal abnormality (1). Myocardial uptake can be intense in the fed state, when the serum glucose and insulin levels are high (Figures 7D–F). Thus, in oncology, myocardial activity is minimized by fasting for 4–6 h prior to the FDG injection.

### Esophagus

**Figure 8:**
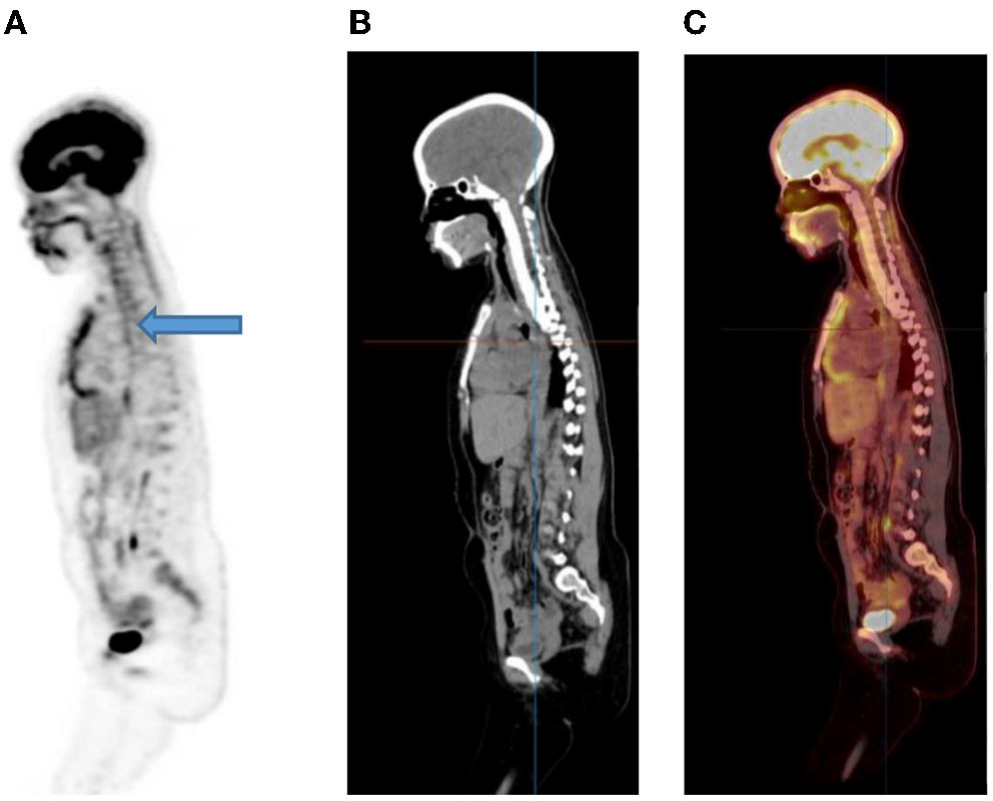
A 15-year-old male known with Hodgkin's lymphoma, referred for a restaging PET/CT post chemotherapy. Sagittal PET **(A)** showing linear uptake anterior to the thoracic spine corresponding to uptake in the esophagus (blue arrow), likely attributed to reflux or esophagitis. The CT **(B)** and fused PET/CT **(C)** will assist in this interpretation too.

**Figure 9:**
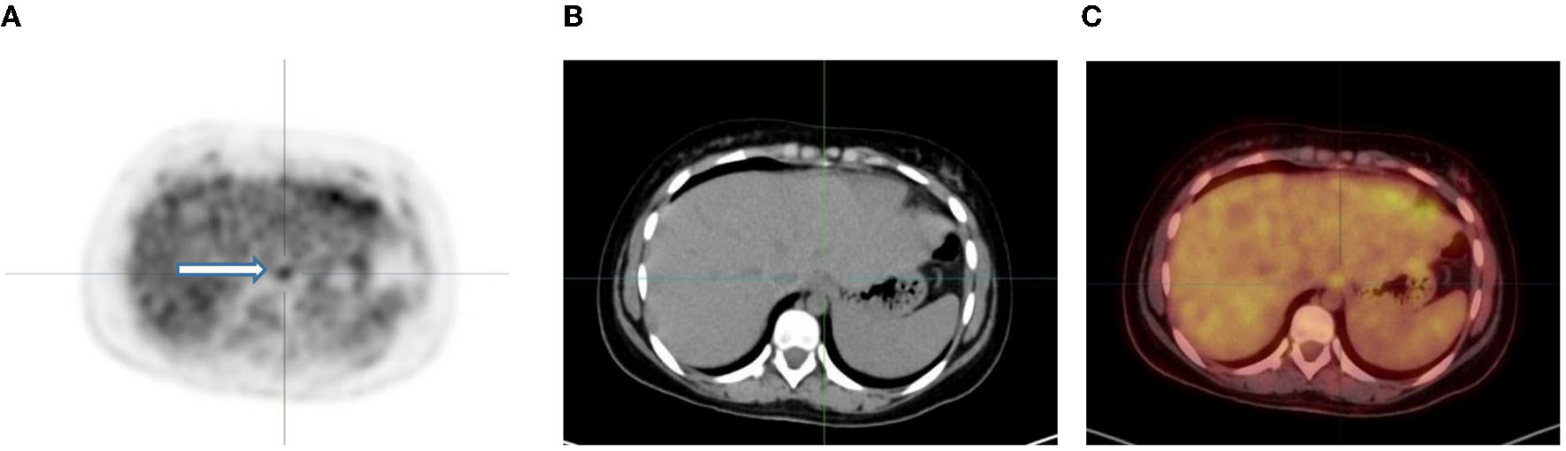
6-year-old female with Ewing's sarcoma of the left proximal tibia. Axial PET **(A)**, CT **(B)** and fused PET/CT **(C)** showing mild diffuse uptake in the gastroesophageal junction (white arrow and crosshairs) that is physiological.

Activity in the esophagus is seen as mild linear uptake anterior to the spine and is best seen in the sagittal plane (Figure 8). Marked uptake along the esophagus is seen in patients with esophagitis from reflux or after radiation therapy. Diffuse and low grade uptake is seen in the gastro-esophageal junction, which is physiological and should not be confused with disease involvement (Figure 9).

## Abdomen And Pelvis

### Gastrointestinal Tract

**Figure 10:**
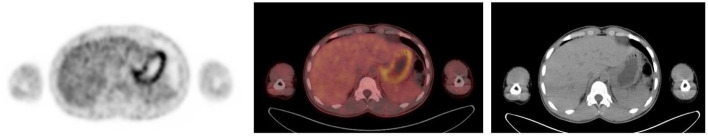
Axial PET, fused FDG PET/CT and CT showing increased FDG uptake along the wall of a contracted stomach, which is physiological.

**Figure 11:**
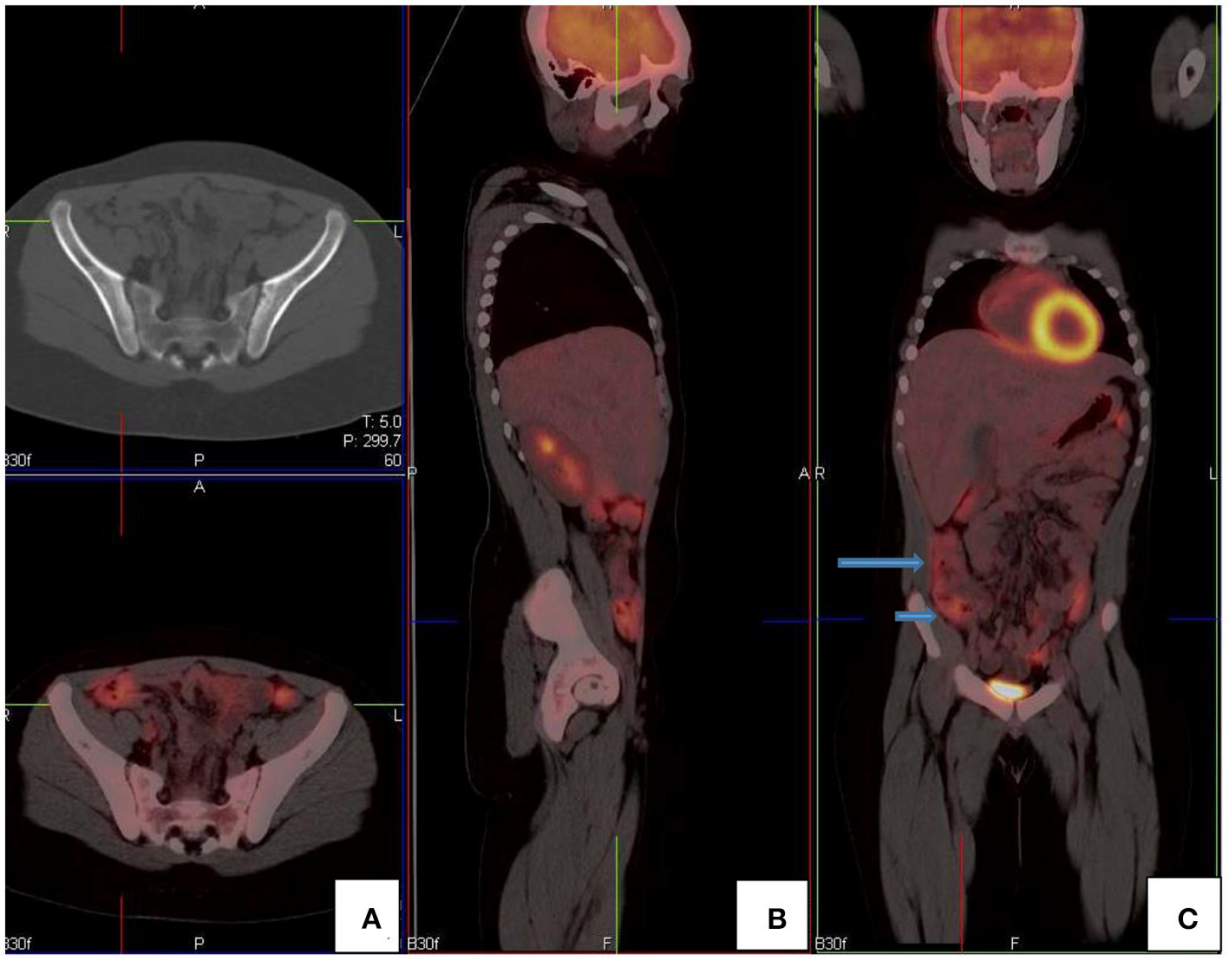
FDG PET/CT **(A)** axial, **(B)** sagittal and **(C)** coronal images show markedly increased FDG uptake in the normal right colon (long arrow). Note the more intense uptake in the cecum due to the higher amount of lymphoid tissue (short arrow).

**Figure 12:**
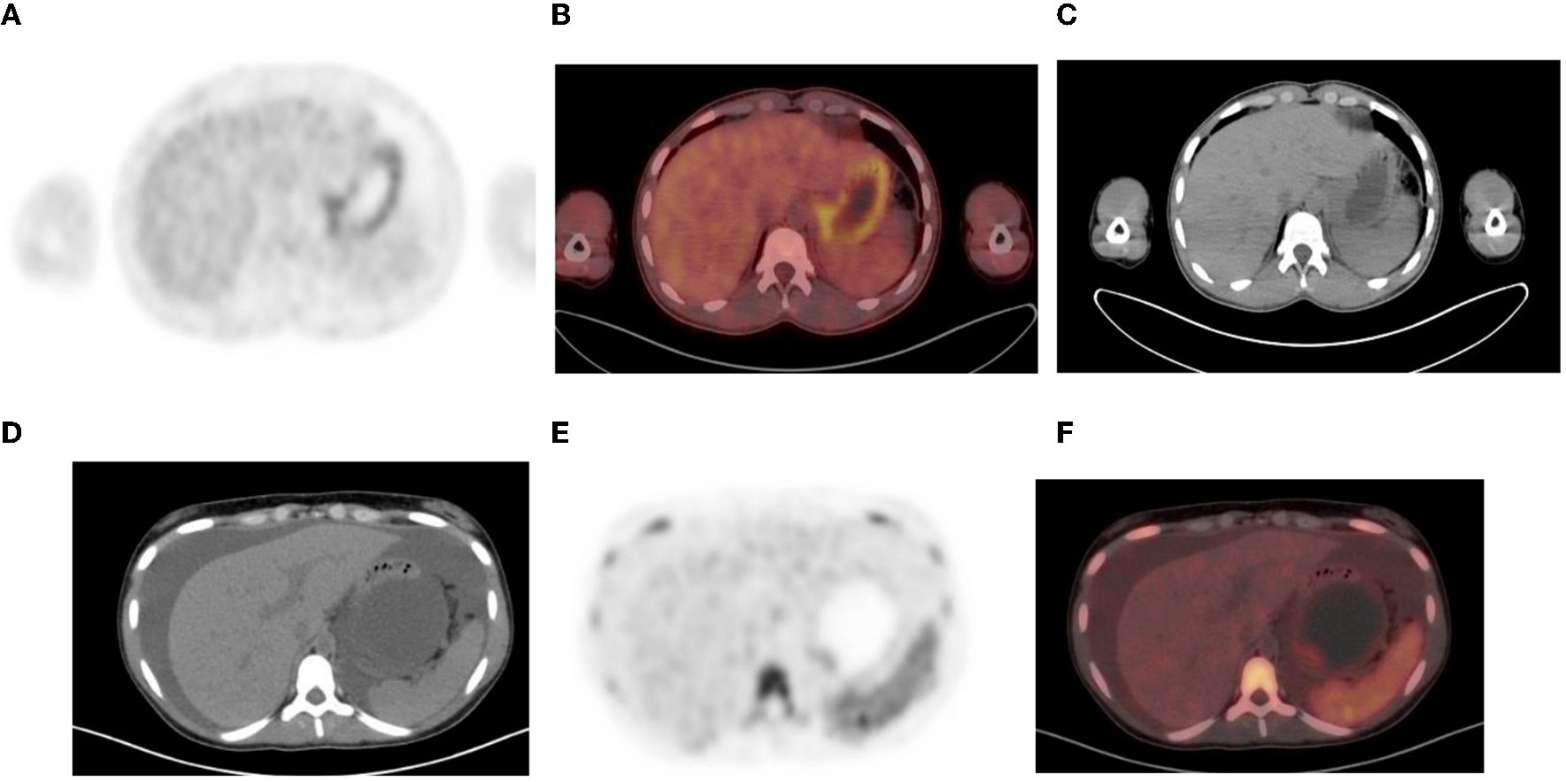
**(A–C)** Normal physiological liver to spleen ratio. Physiological liver uptake is always more than the spleen uptake, except in certain pathologies like lymphomatous involvement of the spleen, HIV or in patients who are post chemotherapy or after the administration of granulocyte colony stimulating factor. Note the physiological the uptake in the gastric wall in a contracted stomach. Note that in **(A)** and **(C)**, non-contrast CT has a limited sensitivity in showing splenic lesions, however the uptake in the spleen is greater than the physiological liver uptake and the presence of splenomegaly is compatible with pathologic involvement. **(D–F)** Diffuse increased uptake in the spleen, greater than the physiological liver uptake in a 10-year-old female with diffuse large B cell lymphoma (DLBCL) prior to chemotherapy. Staging PET/CT showed stage 4 disease with nodal disease above and below the diaphragm, bone marrow and splenic involvement. **(B)** PET and **(C)** fused PET/CT showing increased uptake in the spleen and bone marrow compared to the physiological uptake in the liver. Note that in **(F)**, a non-contrast CT has a limited sensitivity in showing splenic lesions, however the uptake in the spleen is greater than the physiological liver uptake and the presence of splenomegaly is compatible with pathologic involvement.

In the gastrointestinal tract FDG uptake is highly variable and can range from mild to intense uptake, with a focal, diffuse, or segmental distribution. The mechanism of FDG uptake in the gastrointestinal tract is not fully understood and is likely multifactorial. It is attributed to active smooth muscle, active mucosa, secretions that are swallowed, or due to microbial uptake (1). Usually, there is curvilinear homogeneous uptake that corresponds to the gastric wall. A round area of moderate uptake may be seen if the stomach is contracted (1) (Figure 10). Intense uptake may be seen with Helicobacter pylori infection. Uptake in the small bowel is usually low grade but variable. The uptake in the large bowel is highly variable and may be intense, and affects all or part of the colon. The most prominent uptake is seen in the ileocaecal junction than in the rest of the colon, due to a larger concentration of active lymphoid tissue in the ileocecal region (Figure 11). Intense uptake in the cecum may make differentiation of malignancy and inflammation and from a normal variant difficult to differentiate. There is homogeneous moderate uptake in the liver, with relatively less intense uptake than in the spleen (Figures 12A–C). Attention must be paid to the liver to splenic ratio in patients with lymphoma as the reverse (splenic uptake greater than liver uptake) at baseline indicates splenic involvement with tumor (Figures 12D–F).

**Figure 13:**
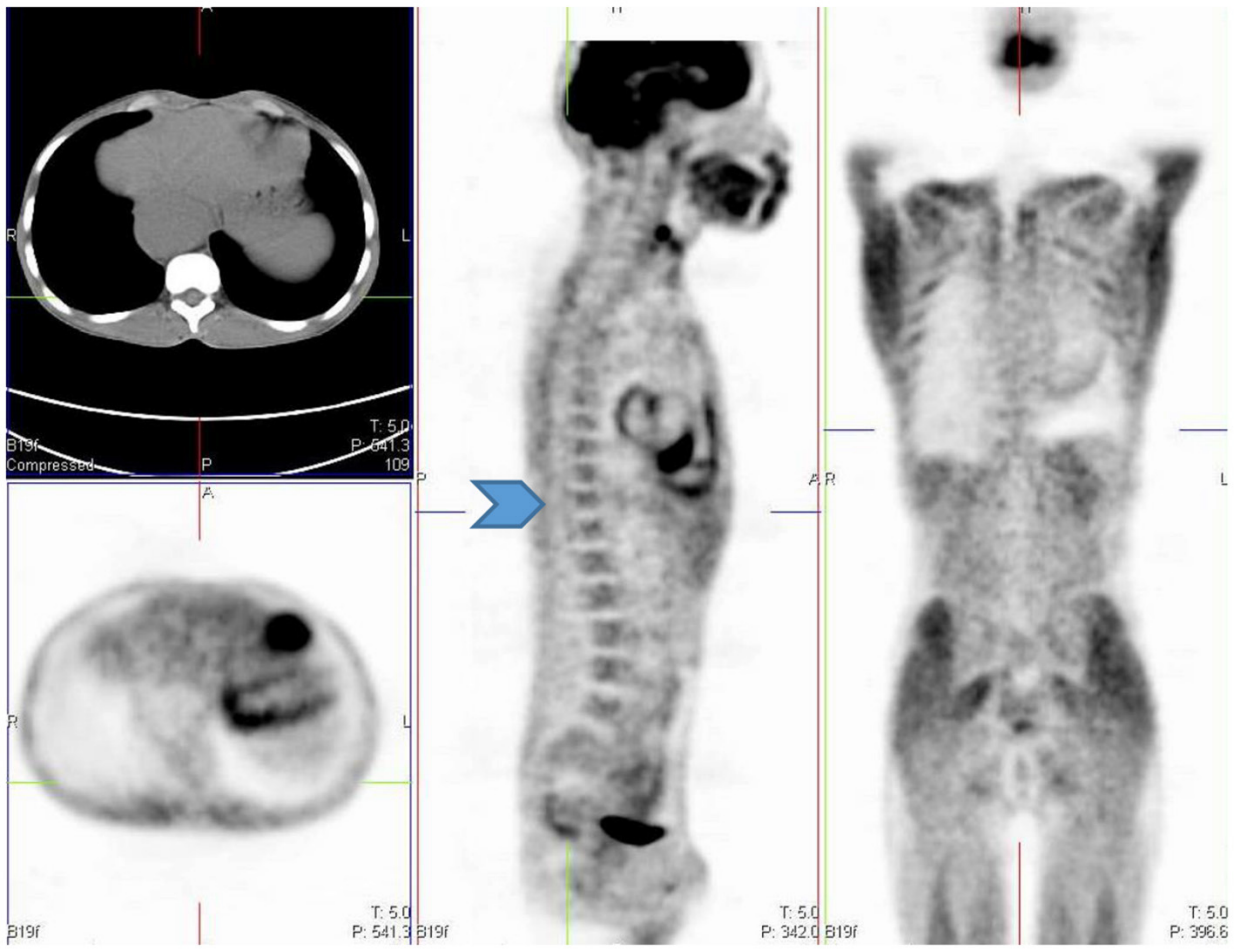
12 year old female referred for evaluation of active vasculitis. Incidental finding of linear uptake in the conus medullaris at T11-L1 level is physiological (Blue arrowhead). Also note the diffuse uptake in the muscles of the chest and lower limb due to playing sport the day before the injection. Other causes of diffuse muscle uptake may include high glucose level prior to injection of FDG.

Low grade, diffuse FDG uptake is commonly seen in the conus medullaris in children and is physiological (2). This is seen as linear or focal increased uptake in the spinal canal and can be seen anywhere from the cervical region up to the lumbar spine on the sagittal and coronal planes (15, 16). It is imperative that all the reconstructed views are viewed to conclude this physiological uptake pattern of FDG (Figure 13).

### Genitourinary System

**Figure 14:**
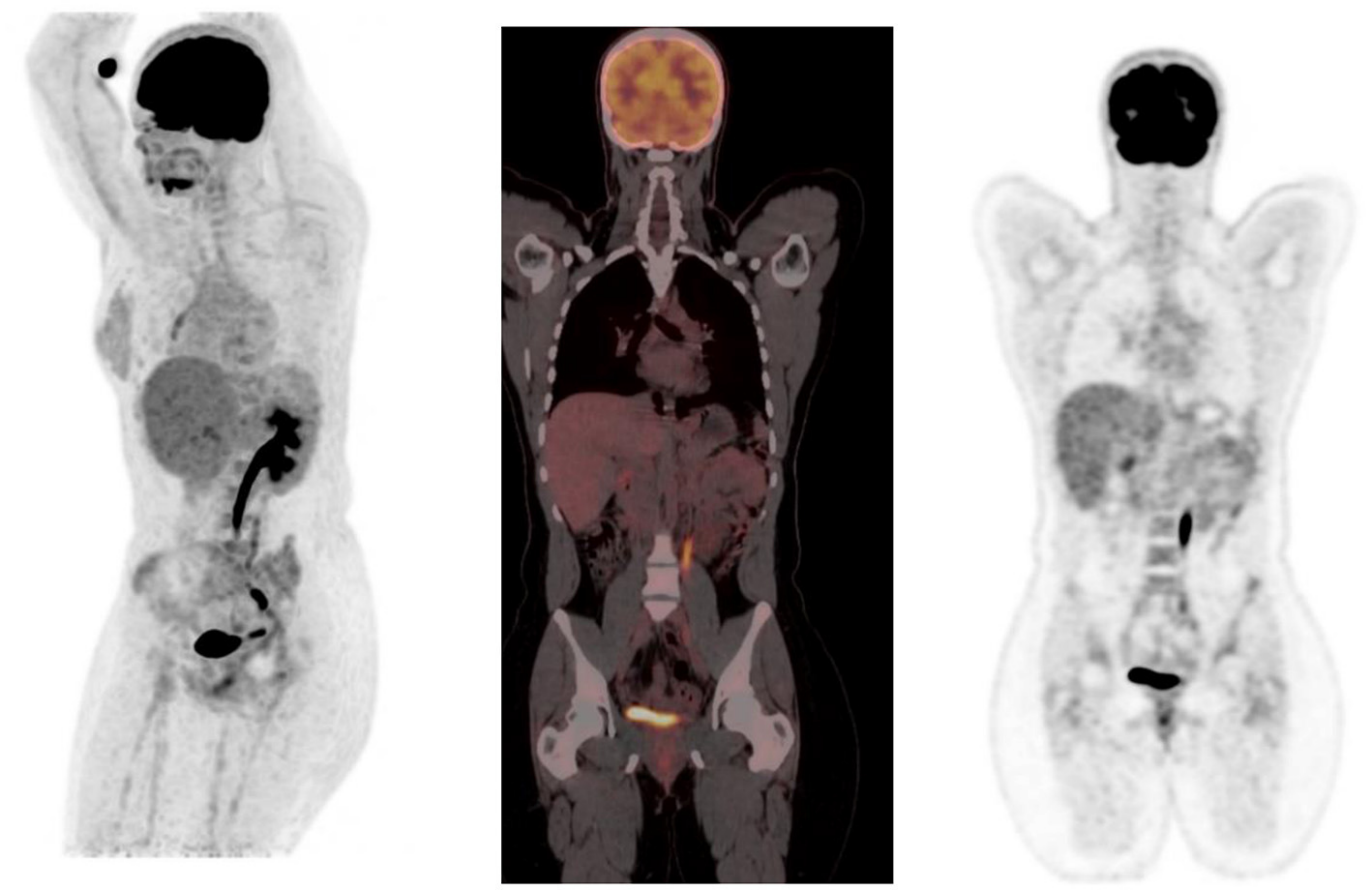
MIP and coronal FDG PET/CT and PET images showing physiological activity in the left renal collecting system, left ureter and urinary bladder. The MIP image is helpful in differentiating tracer excretion in the ureter from nodal pathology. Identifying the uptake to the ureter on CT also helps in differentiating from nodal pathology.

FDG is unlike glucose and is excreted through the urinary system. This results in large amount of activity in the urinary tract from the kidneys and includes the bladder (Figure 14). Reconstruction artifact may occur if there is significant retention in the renal collecting system resulting in interference with the visualization of the upper abdomen (1). The activity can minimized by ensuring the patient is well-hydrated and/or after the administration of diuretics (1, 2). The patient should void prior to imaging if toilet trained, as this will assist with the clearance from the urinary tract. Tracer activity in the ureters can be identified by the anatomical contours of the ureters and with correlation with the CT component of the study. Catheterisation may be necessary in a few cases where the accumulation of tracer in the urinary tract interferes with the evaluation of known lesions close to the genitourinary tract (1). Focal activity within the ureter can mimic malignant lymphadenopathy (1). Urine activity in bladder diverticula may resemble lymphadenopathy or ovarian tumors (1). Other causes of false-positive findings include congenital variants such as ectopic kidney, horseshoe kidney and anatomical distortion due to urinary diversion (1).

**Figure 15:**
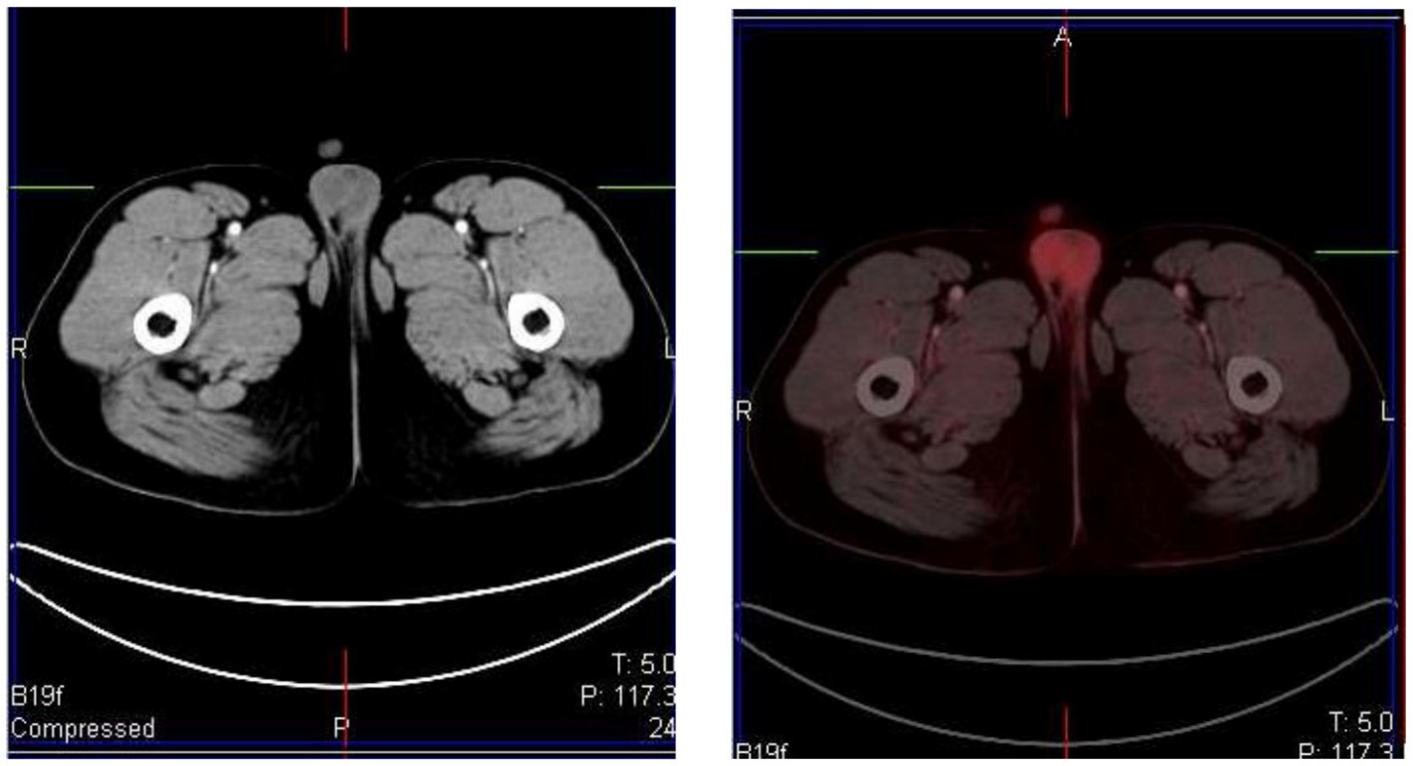
A 16 year old male with Hodgkin's lymphoma referred to evaluate the end of treatment response to chemotherapy. Pubertal boys may have diffuse, increased and symmetrical uptake in the testes. No abnormality was seen at CT.

Testicular uptake in males show a diffuse symmetric pattern with moderate intensity that decreases with age (1) (Figure 15). Males <10 years of age usually show low or no FDG uptake in the testes (17). The uptake in the uterus and ovaries vary with the phase of the menstrual cycle in female children post menarche. Physiological endometrial uptake may be seen during ovulation and menstruation (2). Physiological intense FDG uptake may be seen in the ovaries and should not be mistaken for a nodal pathology in the pelvis (17). A thorough history with the correct phase of the menstrual cycle will help to differentiate physiologic uptake in the ovaries from pathological uptake.

### Musculoskeletal

Muscle uptake is usually low at rest, however physiological muscle uptake can be seen when there is excessive muscle activity post FDG injection or a few days before the study. The uptake is usually symmetrical in the various groups of muscle. Asymmetric and marked uptake in the muscle can occur, especially after excessive exercise or muscle tension. If the patient has been talking during the uptake phase this may result in laryngeal muscle uptake. In babies who suck pacifiers during the uptake phase there may be uptake in the masseter muscles (1). Children who cry during the uptake phase may have uptake in the diaphragm, the crura, and the intercostal muscles. Insulin or recent food intake causes diffuse skeletal muscle uptake, since insulin facilitates FDG into skeletal muscle. Uptake in the muscles may be due to a non-fasted state as a result of the increase in glucose uptake which is mediated by translocation of GLUT 4 from intracellular vesicles into the plasma membrane due to the effect of insulin. In the same manner, glucose metabolism is increased in skeletal muscle during physical exercise. Muscle relaxants such as benzodiazepines can be used to decrease muscle uptake (1). Patients should rest comfortably during the uptake phase to avoid marked muscle uptake.

### Brown Adipose Tissue

**Figure 16:**
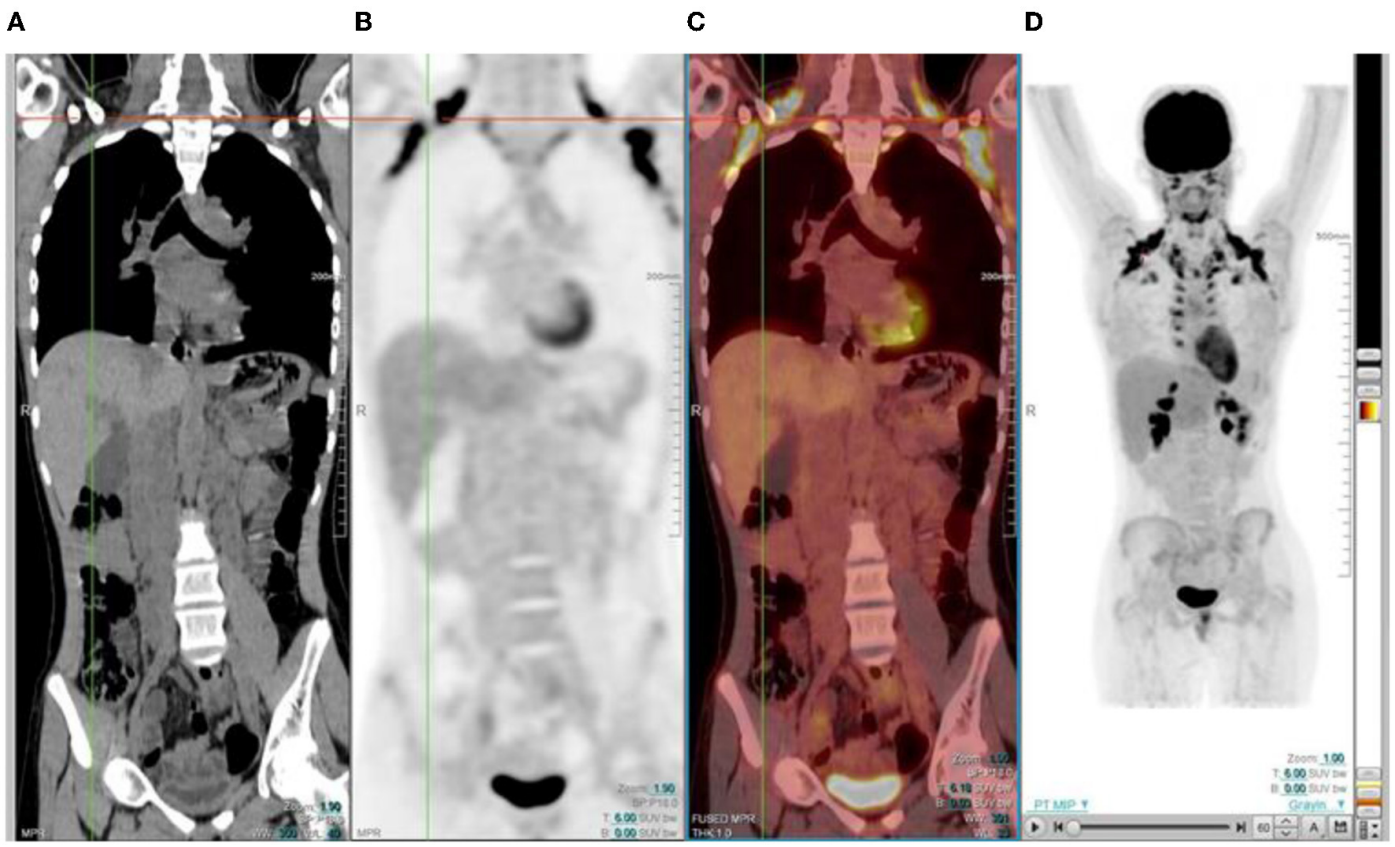
15 year old male with stage 3 Hodgkin's lymphoma referred for follow up FDG PET post chemotherapy. Coronal PET **(B)**, and PET/CT **(C)** showing bilateral symmetrical uptake in the neck and paravertebral region of the cervical and thoracic spine. Crosshairs localized to fat density on CT **(A)**. FDG uptake in brown adipose tissue is commonly seen in children and adolescents. Many pediatric patients have mild brown fat uptake in the neck or supraclavicular regions. However, intense uptake may include pericardiac and perirenal brown adipose tissue. ^18^F-FDG uptake in activated brown fat may obscure sites of pathologic FDG uptake and decrease confidence in the interpretation of the study.

Brown fat is a sub-type of adipose tissue that is rich in mitochondria and helps to regulate the body temperature by non-shivering thermogenesis (17). Sympathetic stimulation helps to generate heat in the body. It is activated in cold temperature, and is usually seen in winter. Physiological and intense uptake can be found in various sites, including the neck, supraclavicular area, axillae, mediastinum, paravertebral and perinephric regions (1, 2) (Figure 16). FDG uptake in brown fat is normally bilateral and symmetric, however, focal and/or asymmetrical uptake can occur, that may result in false-positive findings and misinterpreted as pathology (18). It is helpful to identify this pitfall by localization on CT to a fat density and the lack of any corresponding soft-tissue mass (11). As brown fat is sympathetically innervated, an anxious patient at the time of the FDG injection may contribute to its visualization (11). Data suggests that FDG uptake in adipose tissue occurs more often due to an acute response to cold weather instead of the prolonged period of cold weather (17). Keeping the patient in a warm environment prior to the injection and post injection is a simple approach that is routinely used. FDG uptake due to activated brown fat can also be avoided by the administration of premedication including, diazepam, fentanyl, or propanolol prior to injection (1, 2, 16, 17).

### Growth Plates

Physiological FDG uptake is seen in the growth plates of pediatric patients (2). This uptake may differ during different stages of development and corresponds to the age and the site of the growth plate (2). The highest uptake is seen in the distal femur and looks like a horizontal band of increased FDG uptake in the physes and apophyses (2). The uptake in the growth plates is usually bilateral and usually symmetrical.

### Bone Marrow

**Figure 17:**
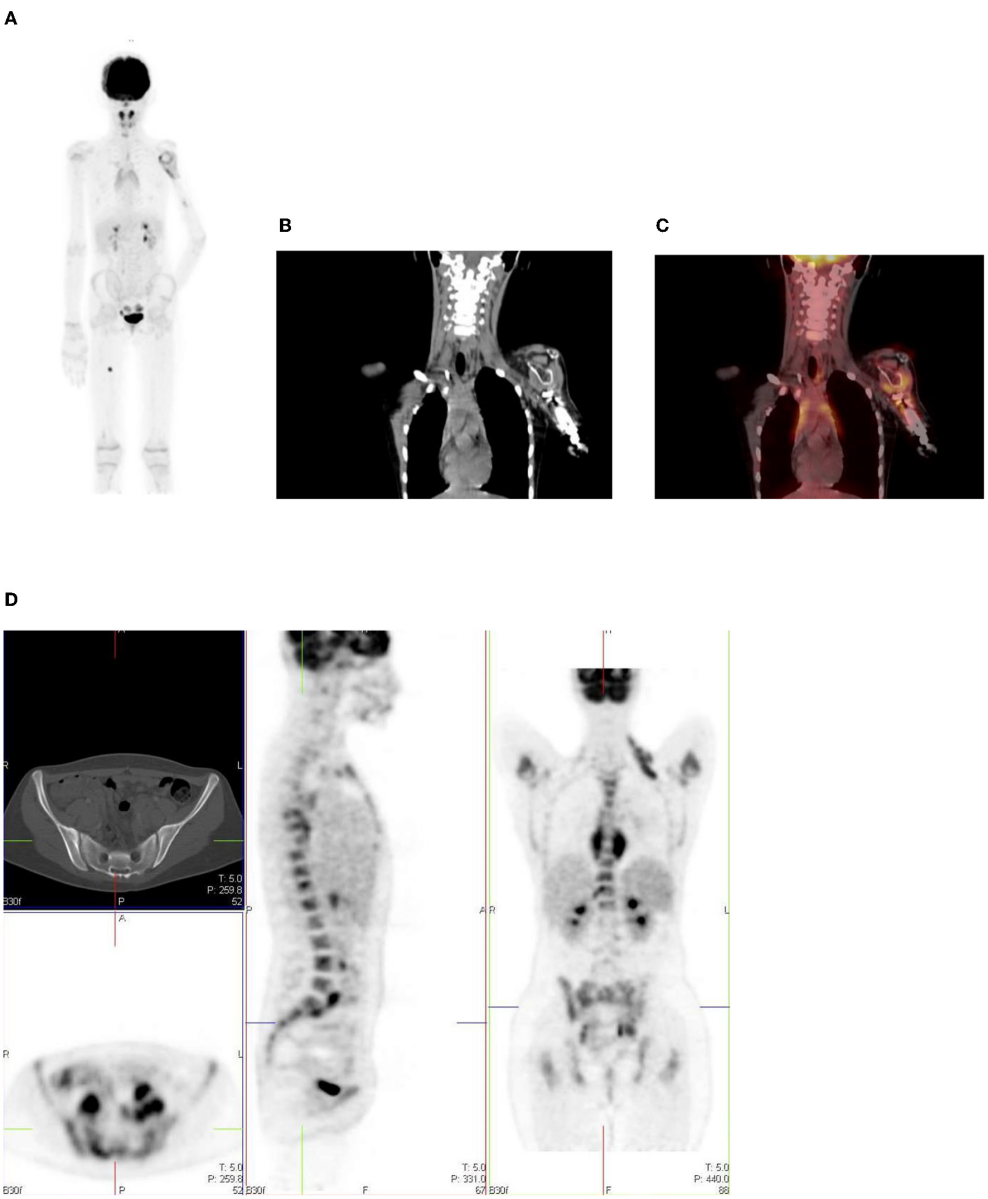
**(A–C)** 9 year old female with high grade osteosarcoma. Coronal PET image **(A)** shows normal pediatric red marrow distribution. There is homogenous FDG uptake in the proximal humeri, proximal and distal femurs, and proximal tibias reflecting normal FDG uptake in red bone marrow. This uptake is usually minimal or absent in adults due to the conversion of red marrow to yellow marrow, which is less metabolically active. Pathology in the left shoulder with increased FDG uptake in the left humeral head and proximal humerus is compatible with orthopedic hardware **(B)**. CT image **(B)** showing the prosthesis and fused PET/CT image **(C)** showing the peri-prosthetic FDG uptake. Note however, active disease cannot entirely be excluded. Due to pain in the left arm, the patient was unable to extend and rotate the left hand for correct imaging position. **(D)** 16 year old female with newly diagnosed Hodgkin's lymphoma. Staging PET shows increased uptake in the marrow, greater than the liver with areas of inhomogeneous and focal uptake, indicating bone marrow infiltration. The CT did not show anatomical lesions (Top left). Note the left neck (site of biopsy confirmed disease) and intrathoracic nodal disease.

Homogenous and low grade uptake is seen in the bone marrow and is less intense than the physiological liver activity. Bone marrow uptake that is more intense than the physiological liver activity is considered abnormal. Increased bone marrow uptake can be seen following chemotherapy, due to physiologic regeneration, and usually resolves within a month (1, 2) (Figure 16). Hyperplasia and hematopoietic stimulation from anemia may also cause an increase in uptake in the marrow (1, 2). Treatment with cytokines such as granulocyte colony-stimulating factor (CSF), hematopoietic growth factor, or erythropoietin can produce diffuse skeletal FDG uptake. Increased uptake can persist for 3 weeks after the discontinuation of granulocyte CSF treatment, therefore, it is advisable to postpone the study until about 4 weeks after the treatment (1). Metastases originating in bone marrow can be distinguished by focal and increased uptake with non-uniform distribution of FDG (14). The red marrow in the pediatric population is metabolically active compared to the yellow marrow as seen in adults. This shows an increase in the uptake in the proximal humeri, proximal and distal femurs and proximal tibias, which are sites of red marrow in a growing child (2) (Figures 17A–C). This should not be mistaken as bone marrow involvement by disease. Reduced bone marrow FDG uptake can be seen several months after external beam radiation therapy and is due to the replacement of bone marrow by fatty tissue (1).

In our institution, interim FDG-PET/CT is performed before the next chemotherapy cycle, and at least 10 days from the previous cycle. The end of treatment PET following completion of treatment is carried out at 6–8 weeks. It is common to visualize a diffuse increased splenic uptake due to recent chemotherapy or treatment with a colony-stimulating factor, and in such scenarios, there is also an increase in bone marrow uptake. Focal increased and inhomogenous uptake in the bone marrow and/or corresponding to an anatomical lesion on CT is in keeping with marrow/osseous involvement (19, 20) (Figure 17D).

### Infection and Inflammation

FDG is not specific for malignancy and is seen in infection, inflammation and benign pathology (1). In inflammation, there is upregulation of lymphocytes, macrophages and granulocytes, which have increased glucose transporter proteins, GLUT 1 and GLUT 3 and thus higher affinity to FDG. Focal FDG accumulation in the gluteal muscles are injection site granulomas, especially in patients with recent intramuscular injection. Patients receiving subcutaneous injection of low molecular-weight heparin can develop FDG-avid subcutaneous areas of soft-tissue attenuation at the injection sites. The accumulation of inflammatory cells and macrophages within these lesions explains the FDG accumulation in the lesions. These nodules can mimic metastatic tumor deposits and the clinical information is imperative to avoid misinterpretation. Soft tissue stranding will guide the inflammatory nature of these lesions. Active vascular inflammation may demonstrate FDG uptake. FDG PET/ CT can help detect large vessel vasculitis and reflect the distribution of active inflammatory activity in the wall of the vessel (1).

### Technical Artifacts

**Figure 18:**
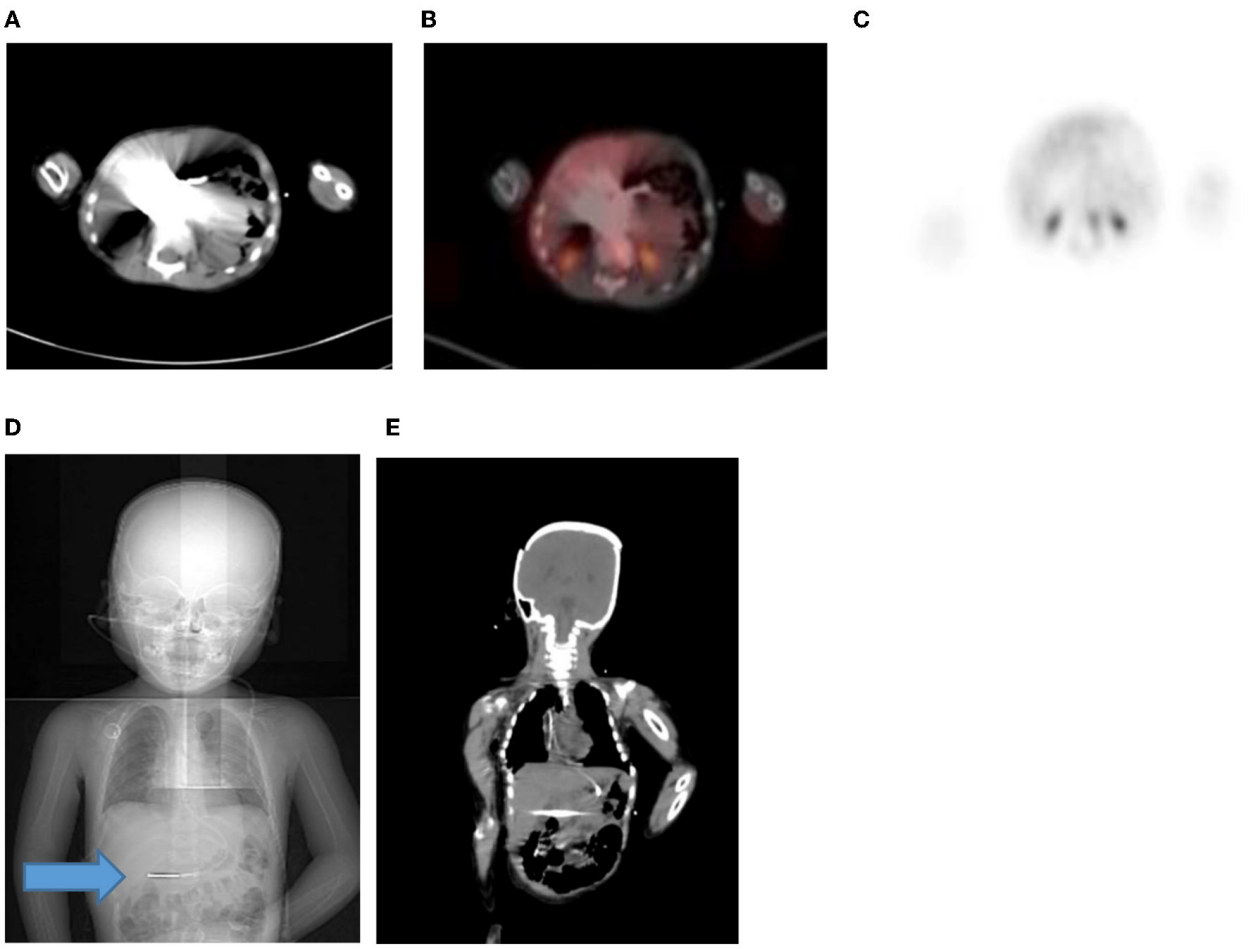
**(A–C)** 2 year old male with neuroblastoma who had a staging PET/CT. Artifact in the abdomen due to metal component of nasogastric tube *in-situ* resulting in streak artifact on CT **(A)** and fused PET/CT **(B)**. Note the PET only image shows that there is normal excretion in the renal calyces with physiological low grade uptake in the liver. Coronal CT scannogram **(D)** shows the tube extending into the abdomen (Blue arrow) with streak artifact on coronal CT **(E)**.

Hybrid PET/CT imaging can create artifacts associated with the CT data rather than PET data for attenuation correction. Increased uptake due to metallic objects such as prostheses, pacemakers, or chemotherapy catheters may cause false-positive findings (1) (Figure 18). The high CT attenuation values results in a false increase in PET attenuation coefficients, creating an overestimation of the PET activity corresponding to the metallic object on the attenuation-corrected image (1). In the same scenario, increased concentration of intravenous and oral contrast leads to the overcorrection of activity and false-positive results at PET if contrast-enhanced CT data are used for attenuation correction. The non-attenuated corrected PET images should be viewed to distinguish this attenuation correction artifact from true increased uptake, as the increased activity will not be seen on the non- corrected image (1).

The ideal manner for determining the SUV in children may be different from that used in adults because of the growth that occurs in childhood. Therefore, in pediatric patients, SUV calculated by body surface area is a better metabolic marker of activity rather than that calculated based on body weight (1).

Respiratory motion causes mis-registration of PET and CT data that can result in attenuation correction artifact that is seen near the diaphragm and base of lung (1).

Injection of radiotracer into central lines should be avoided as the retention of tracer in the line or at its tip can mimic FDG-avid pathology and may be misinterpreted as a false positive finding. The line should be flushed with an ample volume of normal saline if a central line is used for tracer injection due to difficult venous access (1).

Negative scan findings cannot exclude small or microscopic malignant involvement. Tumors with low metabolic activity or poor cellular composition may show minimal uptake of FDG (1). The standardized uptake values do not help to differentiate benign vs. malignant etiology.

Pitfalls related to therapy should be remembered, such as drug-induced pulmonary toxicity. Usage of cytotoxic and immunotherapy drugs may cause lung toxicity with features of alveolar-interstitial infiltrates and alveolar damage, which may have increased FDG uptake (2). Knowledge of the adverse-effects of the cytotoxic drugs and their imaging features will be helpful in the proper interpretation of the images. Post-treatment radiation inflammatory changes are FDG avid areas of consolidation and are differentiated from pathology due to the sharp demarcation from the normal lung, limited to the radiation field (2).

Benign bone lesions show variable FDG uptake in the growing skeleton. Examples are fibro-osseous defects like non-ossifying fibromas and fibrous cortical defects and they appear as focal bone lesions with moderate to intense FDG uptake in first and second decade of life (2). They are asymptomatic lesions and are mostly incidental findings. These lesions are benign, and do not require further investigation or treatment. They are located in the metaphysis around the knee-joints, and are usually oval, radiolucent lesions with a thin sclerotic rim and lie parallel to the long axis of the bone (2).

### Radiation Safety

The main focus of radiation safety is dose reduction, which implies a lower potential risk of cancer (21). Radiosensitivity is increased in children due to actively developing organs at the time of the scan and their longer post exposure life expectancy. Children have a higher relative risk of leukemia, brain, breast, skin, and thyroid cancers compared with adults exposed to radiation post-adolescence (21). To address the concern of radiation burden in children, adult PET/CT acquisition protocols are modified in order to minimize the radiation dose delivered; however, it is imperative to ensure that such modifications do not compromise the diagnostic information required.

## Conclusion

FDG PET/CT is now increasing used in the oncologic evaluation of pediatric patients. The physiological distribution of FDG uptake in children differs from adults and it is important to recognize the normal bio-distribution of FDG in children and take cognisance of the artifacts, and potential pitfalls. Knowing these potential causes of misinterpretation can increase the accuracy of interpretation, decrease the number of unnecessary follow-up studies and improve treatment outcome.

## Author Contributions

All authors listed have made a substantial, direct, and intellectual contribution to the work and approved it for publication.

## Conflict of Interest

The authors declare that the research was conducted in the absence of any commercial or financial relationships that could be construed as a potential conflict of interest.

## Publisher's Note

All claims expressed in this article are solely those of the authors and do not necessarily represent those of their affiliated organizations, or those of the publisher, the editors and the reviewers. Any product that may be evaluated in this article, or claim that may be made by its manufacturer, is not guaranteed or endorsed by the publisher.
